# Comparing Breast Cancer Experiences and Quality of Life between Lesbian and Heterosexual Women

**DOI:** 10.3390/cancers13174347

**Published:** 2021-08-27

**Authors:** Maya Borowczak, Marie C. Lee, Emily Weidenbaum, Anne Mattingly, Anne Kuritzky, Gwendolyn P. Quinn

**Affiliations:** 1 Rowan University School of Osteopathic Medicine, Stratford, NJ 08043, USA; 2 H. Lee Moffitt Cancer Center and Research Institute, Tampa, FL 33612, USA; Marie.Lee@moffitt.org; 3 Department of Obstetrics and Gynecology, Grossman School of Medicine, New York University, New York, NY 10016, USA; Emily.Weidenbaum@nyulangone.org (E.W.); Gwendolyn.Quinn@nyulangone.org (G.P.Q.); 4 Hendricks Regional Health, Danville, IN 46122, USA; Anne.Mattingly@hendricks.org; 5 Trihealth Cancer Institute, Cincinnati, OH 45242, USA; Anne_Kuritzky@trihealth.com

**Keywords:** breast cancer, lesbian, sexual minority, reconstruction, breast cancer support system

## Abstract

**Simple Summary:**

While issues related to support for women with breast cancer have been well studied among heterosexual women, less is known about the supportive care needs of women who are in same-sex or lesbian relationships. Aside from being at increased risk for development of, and mortality from, breast cancer compared to their heterosexual counterparts, there is a growing collection of literature that suggests that lesbian women with breast cancer have different psychosocial and supportive care needs than heterosexual women. The purpose of this study was to examine heterosexual and lesbian women breast cancer survivors’ perceptions of their cancer care experience and support sources. As survivorship care continues to evolve, it is important to recognize not only the specific needs of lesbian minority women, but also the many strengths of this community as these factors may inform future interventions and approaches to improved survivorship care.

**Abstract:**

Background: While breast cancer among women in general has been well studied, little is known about breast cancer in sexual minority women (SMW). Aside from being at an increased risk for development of, and mortality from, breast cancer compared to their heterosexual counterparts, there is a growing collection of literature that suggests that SMW experience breast cancer differently to heterosexual women. Methods: Qualitative study of both straight and lesbian women with a diagnosis of breast cancer. Focus groups were conducted to assess straight and SMW experiences pertaining to perceived barriers, resources/support from partners as well as attitudes pertaining to breast reconstruction. Results: A sample of 15 participants (10 straight and 5 lesbian women) were included in the present study. Focus group themes focused on support, wishes for support, satisfaction with inclusion of partner, fear, perceived discrimination, quality of life, body image, treatment delay, financial concern, frustration with the system, reconstruction, access to information, and attitudes towards cancer diagnosis. A majority of women in both groups chose to undergo breast reconstruction. Conclusion: In our study, SMW experienced their breast cancer treatment through a uniquely supportive and positive lens, often with higher relationship satisfaction and better self-image when compared to straight women.

## 1. Introduction

Breast cancer is the most commonly diagnosed cancer among women in the United States and is the second leading cause of cancer death in women after lung cancer. Approximately 1 in 8 women will be diagnosed with invasive breast cancer in their lifetime [1].

While breast cancer among women in general has been well studied, little is known about breast cancer in sexual minority women (SMW) as data on sexual orientation and same-sex partnerships are seldom recorded in medical practices and cancer registries [2].

SMW are at an increased risk for development of, and mortality from, breast cancer compared to their heterosexual counterparts due to disproportionate exposure to certain risk factors [3]. Recently, ASCO guidelines and the NIH have classified sexual and gender minority (SGM) people as a group experiencing health disparities [4,5]. Additionally, quality of life (QoL) is an important aspect of cancer survivorship and can be related to the quality of the cancer care experience. However, little is known about gay/lesbian women cancer care experiences and quality of life. Aside from harboring an increased risk, there is a growing collection of literature that suggests that SMW (lesbian women in particular) experience breast cancer differently to heterosexual women [2]. Factors such as LGBT identity disclosure and corresponding stressors associated with being of minority group status, along with unique supportive networks, may play a role in this difference [2,3].

While social support appears to be significantly associated with cancer progression and outcome, especially for female patients with breast cancer, little is known about the supportive relationships in SMW [6]. Findings from studies on married, heterosexual couples have suggested a link to earlier breast cancer diagnosis and decreased rates of breast cancer-related mortality [7]. However, it is unclear whether these findings in heterosexual women can be generalized to SMW. Normalization of SMW identities and the continued failure to collect sexual orientation and gender identity data in health care settings may contribute to a lack of understanding of the specific needs of the sexual minority population and may lead providers to assume that the existing, heteronormative social supports are sufficient to address the needs of SMW breast cancer patients [8]. These heteronormative assumptions (presumptions of heterosexuality as the default sexual orientation) may manifest in considerable pressure for SMW to choose reconstruction from breast cancer survivor organizations and from within the medical system.

Sexual or gender identity may be an important factor influencing treatment and reconstruction choices, but research regarding breast reconstruction choices amongst SMW has been limited [9].

As the number of cancer survivors and caregivers continues to grow, it is increasingly important to understand the nuances that may exist along a variety of dimensions, including sexual orientation [7]. The purpose of this study was to examine straight and lesbian women breast cancer survivors’ perceptions of their cancer care and current supportive resources. Additionally, this study aimed to compare straight and lesbian women’s thoughts regarding breast reconstruction, as well as their perception of social support and its potential role in making those decisions.

## 2. Materials and Methods

Participants were recruited by clinicians at the cancer center through word of mouth and distribution of flyers in the clinic. Additional efforts to recruit women who identified as lesbian were made through LGBTQ community events and support groups. Women who met the following criteria were offered to participate in this study: breast cancer survivors who identify as LGBTQ; prior diagnosis of breast cancer; not currently on active treatment; had been offered breast reconstruction as part of their treatment plan. A gift card was provided to all participants at the conclusion of each focus group. This study was approved by the University of South Florida Institutional Review Board #Pro00023886 (27 February 2016).

### 2.1. Demographics

Participants who agreed to be in this study completed a demographic form survey. Twenty-three questions assessed demographics including age, sex assigned at birth, race/ethnicity, marital status, employment status, household size, household income, education level, gender identity, sexual orientation, location of breast cancer treatment, age at diagnosis, year of diagnosis, stage at diagnosis, health insurance status at time of diagnosis, date of last normal mammogram, type of cancer surgery received, type of breast reconstruction received or not, whether they received chemotherapy, radiation, or hormonal treatment, and if they used fertility preservation prior to cancer treatment.

### 2.2. Focus Group

The focus group discussions followed survey completion and were facilitated by two moderators and were approximately one hour in length. Using a semi-structured interview guide, participants were asked to discuss their thoughts and experiences on the following topics: perceived barriers at the time of and throughout the course of treatment (misinformation, communication, timeliness or administrative issues), resources and support (trust in system or provider, comfort discussing questions, effect of experience on relationship with partner and/or on self-image, level of support from partner, family, friends, community, and the quality of available resources), and opportunities for improvement. A total of 6 focus groups were conducted ranging in size from 3 to 6 women per group. A total of 10 straight and 5 lesbian-identified women participated in this study.

## 3. Results

Demographic data and breast cancer treatment information were collected from participants and are summarized in Table 1. A total of 15 women participated in our study, with 10 identifying as straight and 5 identifying as lesbian. The mean age at the time of this study was 56. The mean age at diagnosis was 50. The majority of participants were White and married or living with a partner.

### 3.1. Support

Overall, both lesbian and straight groups reported high levels of support from others.

The majority of straight women avoided any significant mention of their partner’s involvement throughout their care—some indicating more stress and anxiety stemming from more ‘science support’ than emotional support.

“(My husband) is a scientist, so I had science support… created a little bit more anxiety… the emotional support was harder.” (straight woman).

In contrast, all lesbian women reported receiving extraordinary involvement and support from their partners, with one woman indicating an advantage in having a female partner as a caregiver.

“(My partner) couldn’t have been more supportive. No matter what it was, she did it. It was everything- to have the experience it was, it couldn’t have been any better.” (lesbian woman).

“I think one advantage we have is that we have a female partner. Most of the time, they are a much better caregiver. I think there is very much of a maternal instinct for that caring... They would do absolutely anything… I’m not saying that men don’t, but women have a tendency to be able to swallow some things that are not as comfortable in looking at that a man might be more uncomfortable with. Helping to change the bandage, draining it when you have an infection—those kinds of things. You can be real fortunate when it comes to things like that.” (lesbian woman).

Further, lesbian women reported no negative effects on their relationship with their partner, which is in contrast to straight women. Several lesbian women reported developing an even deeper relationship with their partners. Many straight women, in contrast, reported feeling like their relationship became forced and rushed, along with emotional and physical struggles taking a toll on their relationships.

“I feel like before, it was like dating and trying to figure out if we’re going to—meant to be married. And—and with this, you—with cancer you just want to know if it’s “Yes” or “No” because I’m done dating. So, the person’s going to get cut off for or rushed, you know, from—because, I just don’t have direction.” (straight woman).

“It’s the emotional part I struggle with—not being able to be the wife I used to be.” (straight woman).

“There’s that certain amount of apathy when it comes to sex. You have no hormones. That’s why I always say it took 10 years away from me. And here’s my husband who is on testosterone and I’m like, ‘Okay, sex is great and I love it. But if I’m going to have to be the one to say let’s go have sex, it’s not going to happen.’ I don’t have any of those hormones. So for us it’s been a struggle... The dryness and the discomfort. You’ve got all of that on top of it. Who wants that? I think it’s definitely been a huge struggle.” (straight woman).

“But for us, we spent more time together. I feel like we were crazy close. I don’t want to say that this made us closer, but it just gave us a little bit deeper relationship. We just talked about anything.” (lesbian woman).

“I feel very fortunate. (My partner) has stuck by me. There were people there whose husbands took off, wouldn’t touch them, and they had a lot of difficulties.” (lesbian woman).

“Every time October comes up and it talks about breast cancer, (my partner) said, ‘I’m so okay with that because I look at you and I still have you.” (lesbian woman).

The majority of lesbian women expressed a high amount of trust in their providers, while only some straight women reported a similar sentiment.

“They were very open to everything that I presented. They said, “Just tell us what you want to do, and we will work that into what we are planning for you. If there’s something that’s contradictory, we’ll let you know and work it out.” I thought that was phenomenal… Not only were they willing to incorporate my beliefs and preferences into what they felt was necessary medically, but when they saw it was worth something they asked me for my input so they could share it with other people and make other patients more comfortable and happy. I thought that was really neat.” (lesbian woman).

“She was excellent. She was very knowledgeable. When she sat down, she sat down on her stool always across from you. She never acted like she was in a hurry. She just sat there and looked at you and talked to you. Coming into the room and leaving the room she was in a hurry, but while she was with me she didn’t act that way. But I really felt like she didn’t have to open the file. She had already looked at it. She recalled what we had last talked about.” (lesbian woman).

Many straight women expressed being at the hands of science and their doctors throughout their treatment process, with motivations for treatment options often dependent on fear of future cancer re-emergence.

“I didn’t think there were a lot of decisions left up to us. I was at the hands of science and my doctors.” (straight woman).

“Not knowing, you let your doctors guide you. When I got to the diagnosis portion, I didn’t like the surgeon. I didn’t like his bedside manner. That’s when I started making phone calls and someone said, ‘you need to take all of your records and go to (another doctor).’” (straight woman).

Many lesbian women, on the other hand, expressed significant autonomy and confidence in their decisions, irrespective of some women’s desires for increased physician involvement.

“(My doctor said), ‘if I was your mother,’ and I said, ‘you’re not my mother and I will do what I feel is right for myself.’” (lesbian woman).

“Give me all the information that I need to know and I’m going to make a decision like that.” (lesbian woman).

“What I didn’t get was a little more encouragement to think about the consequences of making that (reconstructive) decision. How will you feel later on?... That could help somebody today maybe make a different choice.” (lesbian woman).

### 3.2. Wishes for Support

Straight women expressed a desire for health care providers to ask what they can specifically do for them to help. Others expressed an interest in getting information on how to be a better spouse or partner, or where to get an inexpensive wig. Several women also mentioned feeling overwhelmed and wanting either somebody to talk to them at a peer level and tell them what to expect, or recommendations on resources to turn to for answers.

“There’s different ways people need support, but if you could ask the patient, “We’d love to help you out. What—what is it exactly—we can—we can do our normal generic thing—speech to him, but is there something else we can focus on?” because people are so different.” (straight woman).

“I learned that from somebody in the chair next to me. That’s the information you need to give out.” (straight woman).

“If you’ve never had that experience, you don’t know what to do next. I often thought at the time it would’ve been so beneficial to have somebody that had been through it that could help me and suggest things.” (straight woman).

“Where are some good resources?… It’s so overwhelming. Just maybe a takeaway of what to do next or where I can go to get some answers.” (straight woman).

Lesbian women expressed the necessity of having somebody there that is close enough to them that they can ask for help to do things for them—the small things that may seem insignificant but turn out to be critical in day to day life. 

“It’s all the little stuff that you do day in and day out that you don’t think about until you have to do it and you can’t.” (lesbian woman).

### 3.3. Satisfaction with Inclusion of Partner

Ultimately all of the women who identified as lesbian were highly satisfied with their providers’ inclusion of their partners throughout the treatment.

“She was included in everything. She was allowed to stay in the hospital room with me after my surgeries and everything. It didn’t really matter if it was the surgeon or the janitor, everybody treated us the same way. When they spoke with us—and it was always an us that they spoke with—not only did they speak to me, but they also spoke to her.” (lesbian woman).

### 3.4. Fear

A sense of fear was noted among the majority of straight and lesbian women in relation to recurrence and unanswered questions.

“I got to feel confident, and I don’t, and it’s causing me anxiety.” (straight woman).

Another issue relating to fear that emerged in these discussions was fear of a hereditary component to their cancer and a lack of communication from physicians to discuss or settle those anxieties. Some women expressed a desire for more open communication with physicians, with a greater emphasis on the unique needs of lesbian women.

“Like you, my mom was diagnosed. My mom was gone in less than a year and she was supposed to be fine. So I had a bunch of questions about information that she was provided versus what actually happened. I wanted to know if that was going to happen to me.” (lesbian woman).

“I think the doctors have to be more open to talking with us and maybe asking questions that we never were asked. For us, as lesbians, there is one thing that makes it a little harder. Men can get breast cancer, but it’s a lot more in women. When you have two women and my partner gets breast cancer, it’s scary to me. Wow. It could happen to me. And nobody ever addressed that with us or talked with us about it. I don’t think any of the doctors ever talked with us about how we were feeling as we were going through this.” (lesbian woman).

### 3.5. Perceived Discrimination

Some lesbian women reported feelings of perceived discrimination, but these feelings were generally confined to the first clinical encounter and dissipated afterwards. 

“North Carolina is a whole lot different… there were times when, although they would try not to act any different, you could tell by looking at them or the lack of them not looking in your eyes. There were just times. They never rejected my partner. They allowed her whenever I wanted her to be whenever.” (lesbian woman).

“The medical personnel are great about it. It’s before you get there- the ones who would stop somebody from getting in the door, not once they got in the door.” (lesbian woman).

### 3.6. Quality of Life 

The majority of straight women reported some sense of decreased quality of life and expressed more concerns about side effects such as their experiences with lymphedema, hot flashes and hair loss. No lesbian women expressed concerns about significant side effects in relation to quality of life. 

“I would call it a curse. I swear it took 10 years off my life. I’m almost nine years post and I live with hot flashes. My husband calls me a Freon rat. I don’t come out when summer hits. I’m like a prisoner of my house.” (straight woman).

“17 pages of side-effects. I got every one.” (straight woman).

### 3.7. Body/Self-Image

More straight women reported some sort of negative impact on their sense of femininity and/or body than lesbian women who reported no impact or a positive impact on their self-image and approach to life.

“We’re more stable with ourselves and our self-image.” (lesbian woman).

“You can’t find one that fits (bathing suit) right because you don’t have a normal body. You don’t feel pretty for a long time.” (straight woman).

“I don’t think it’s changed the way I see myself as a woman. I am who I am.” (lesbian woman).

“After the surgery, I was like, ‘man, I need some flannel shirts to hang out in and things that button.’ But other than that, after all that was over, I just went back to wearing everything I had worn before.” (lesbian woman).

### 3.8. Financial Concerns

Lesbian women did not report any issues regarding finances or insurance, while straight women expressed mixed reviews. 

“Everything was paid from start to finish… I had a really good experience on that end of it.” (straight woman).

“I had some trouble, and I had really good healthcare coverage… You’re fighting for your life, yet you’re trying to fight for coverage to pay for things.” (straight woman).

### 3.9. Treatment Delay

In both lesbian and straight groups, there were a few (a minority) reported instances of significant delay in treatment and/or misdiagnosis. Several straight women reported a rather quick process from diagnosis to treatment, whereas several lesbian women were more neutral in their classification—they experienced no actual issues in scheduling or in finding a provider, it just felt long to them.

“It took three months to get from finding a lump to getting a diagnosis—which I think is just ridiculous… So it took from April to July before I actually got the diagnosis, when I knew the whole time what it was.” (straight woman).

“I felt like it was really long. And everybody kept saying—especially my friends that were doctors—“In the medical world, this isn’t very long.” (lesbian woman).

“It does feel like forever.” (lesbian woman).

### 3.10. Frustration with System

A majority of straight women reported experiencing frustration with the system (owing to a variety of factors such as poor bedside manner, and confusion and uncertainty with regard to their future transition into survivorship) compared to lesbian women, where the majority did not explicitly express experiencing any particular frustrations.

“I’m confused by what’s going to happen on Friday when radiation ends. I feel like I don’t have any direction.” (straight woman).

### 3.11. Reconstruction

A majority of both straight and lesbian women chose to have reconstruction (but more lesbian women decided against reconstruction, compared with straight women).

“I opted for the bilateral because I didn’t want to go through the process twice if it came back in the other one.” (straight woman).

“I had reconstruction. If you’re going to have reconstruction or an implant put in, do both. One is north and the other one is south if you don’t.” (lesbian woman).

### 3.12. Access to Information

Both straight and lesbian expressed no difficulties with accessing information—rather indicating an overabundance of resources.

“It was really difficult because you were being given a diagnosis and a lot of information and you wanted to make a decision and you wanted to make it yesterday. You didn’t know the questions to ask.” (lesbian woman).

“(The nurse) was like, ‘if you’re going to check the Internet, here are the only two websites I want you to use for resources.’ I thought that was really helpful. We can all spin out of control on the Internet. But the pamphlets, books, and things were all great.” (lesbian woman).

Several straight women relinquished their personal searches for information and relied on their physicians and the information provided to them in pamphlets. 

“I knew that I just had to put it in their hands because they were the professionals. So, I stayed off the internet.” (straight woman).

“I think the hospital and doctors just helped me. They said to do this and do this and do this.” (straight woman).

Several lesbian women, however, expressed a desire for more complete information so that they could review it themselves, and also so that they could avoid comparing themselves with other cancer patients.

“I want an overload of information. I’d rather you give me more than I would ever need than you just giving me the little pieces that I do need, because I want it all and then I want to pick it out from there.” (lesbian woman).

“When you’re dealing with all the other stress of everything, you’re almost like, ‘Why didn’t they tell me?’ It’s more of an emotional thing than an informational thing.” (lesbian woman).

### 3.13. Attitudes towards Cancer Diagnosis

Many straight women reported feelings of shock and devastation when faced with their diagnosis of breast cancer, while many lesbian women emphasized a greater interest in getting the facts and moving on—many even with a positive attitude with regard to their diagnosis and treatment.

“Just when you’re this young, you feel like everything is stolen.” (straight woman)

“My understanding is that they don’t do this a lot of place, but when I went for—I changed the names of everything. I didn’t like the way chemo sounded, so I went for healing treatments. I didn’t like the way radiation sounded, so I went for love beams. It was all that spin that I put on it.” (lesbian woman).

“I used to say that it’s here—it’s in the car. It’s in the trunk. It’s here. But I’m never giving that fucker the keys. It’s not driving. This is how this is going to go.” (lesbian woman).

“But I really believe that your experience is what you think your experience is going to be. So for me it was just like, “Alright, this is just like having my appendix taken out. Let’s just get it out and move on and I’m done.” (lesbian woman).

## 4. Discussion

In this study, we examined straight and lesbian women breast cancer survivors’ perceptions of their cancer care, supportive resources and breast reconstruction. We identified similarities between both groups in terms of high levels of support from others, feelings of fear in relation to either recurrence and/or unanswered questions, occasional treatment delay and no difficulties with accessing information. Differences were noted in the level of support received from their partners and its impacts on their relationship, along with differences in quality of life and body image.

It has been well documented that partners and caregivers provide important social support for women with breast cancer [10,11,12,13,14,15,16,17,18]. For example, many patients with breast cancer have experienced hopelessness and depression during and after their treatment. Social support provided during this period can lead to increased levels of hope and can decrease rates of depression [19,20,21]. While less is known about these psychosocial impacts on SMW, several studies suggest a comparable importance, with some even suggesting that same-gender partnerships provide unique and possible benefits to SMW [18,22,23,24]. These studies demonstrated that SMW in same-sex partnerships had either equal or more support than heterosexual women in relationships/marriages [18,25]. Our results are consistent with these findings as SMW reported a similar sentiment and greater degree of partner involvement, often citing that the shared female experience provided a deeper connection between the couple.

Furthermore, there has been significant literature describing the quality and stability of patients’ relationships with their partners throughout treatment. It seems that breast cancer survivorship and treatment often have a neutral and protective effect on marriage among straight people, unless there were already marital troubles prior to the cancer diagnosis [26]. Some features of marriage, particularly social support, have shown a consistent and positive impact on early access to cancer detection, treatment and survival care, better adherence to treatment and less psychosocial distress [27]. Limited research has been conducted on SMW’s relationships through breast cancer treatment and survivorship, but our study found that SMW indicated a positive or neutral effect on their relationship with their partners as compared to straight women, who indicated feeling more anxiety and stress in their relationship. This finding differs from several existing studies that found no significant differences in relationships between SMW and straight women [28,29].

Another area of interest in the current literature is whether SMW are satisfied with the emotional support their partner received from medical providers, and with the inclusion of their partner in medical decisions and respect received from the medical staff. Our study demonstrates that SMW have satisfaction with these particular aspects of their care. This is in contrast to a previous study that found higher levels of dissatisfaction with care amongst SMW compared to straight women [30]. Satisfaction may be related to geographic location, in that some areas of the country may be more likely to have cultural sensitive providers with training on providing care for SMW, which may account for differences in satisfaction [31]. However, while all of our focus groups were conducted in Florida, participants were treated in several other states (New Jersey, New York and North Carolina). While several SMW stated experiencing instances of perceived discrimination with respect to geographic location, overall satisfaction was not affected.

While it has been presumed that pervasive heterosexism, stigma and discrimination present a gap in quality of life between SMW and straight women, there is an increasing body of literature that suggests otherwise. Currently, certain theoretical frameworks, like the minority stress model, would suggest that SMW would experience additional burdens due to their already stigmatized identity [32]. However, recent studies have found similar quality of life outcomes between the two groups, postulating that this similarity might reflect SMWs’ alternative response to minority stress [33]. Our study showed similarities between the two groups quality of life, and given SMW’s closeness with their partners, and even enhanced relationship post treatment. It is possible that exposure to chronic minority stress contributes to a heightened resilience that facilitates coping with breast cancer and that SMW may develop mastery of cognitive processes to deal with distress since the development of sexual minority identify is typified by stress, emotional turmoil, and social rejection [29].

In addition to heightened resilience, optimism has been noted to be a strength particularly associated with SMW [34,35,36]. Optimism refers to a more positive attitude and has been suggested as one of the factors that promote health and well-being in LGBTQ populations [34,35,36]. A sense of optimism was expressed by SMW in our study While both straight and lesbian women experienced fear and similar concerns with regard to their cancer diagnoses, lesbian women expressed a slightly more positive outlook and reaction and less avoidance moving forward. They maintained more autonomy, less frustration with the system, and less (or less emphasis on) negative effects on quality of life when compared to straight women.

Similarly, we found that lesbian women in our study reported less negative impact to their body image as compared with straight women. These findings may be related to the general sense that lesbian women’s more positive attitude towards cancer and/or values that generally place less emphasis on external appearance compared to straight women [18,37,38,39,40]. These themes re-emerged in the context of breast reconstruction. While we found a majority of women in both groups choosing to undergo breast reconstruction, more SMW opted against reconstruction compared to straight women. While there is limited research on this, the current literature supports that regardless of surgical choice, SMW seem to perceive their values with respect to their body as different and rooted in their sexual minority identity. It seems that SMW value body strength, survival and physical function over outward appearance with regard to their decision for reconstruction [3,37,40]. Thus, these findings support the idea that among SMW who decided against reconstruction, a similar value system prevailed, manifesting in their specific choices of reconstructive procedure [34].

This study adds to the current limited volume of literature regarding the experience of breast cancer treatment and survivorship in SMW. The qualitative approach adds to its strengths and overall goal of better describing the personal experiences of a small number of SMW. It must be noted, however, that our study is limited by a small sample size, conducted in one city in the South, which makes it difficult to generalize to other populations or draw strong conclusions. Selection bias may have occurred during our attempt to recruit lesbian women as women who are not open about their sexual orientation may not have elected to participate. Additionally, heterosexual women in our study were more likely to receive chemotherapy, which may have affected how they perceived their cancer care. Lastly, our sample lacked racial and ethnic diversity, which impedes our ability to generalize results. As survivorship continues to improve, providers must recognize the unique experience SMW have as they traverse the current landscape of breast cancer treatment.

## 5. Conclusions

Through examining straight and lesbian women breast cancer survivors’ perceptions of their cancer care and current supportive resources, we found that SMW experienced their breast cancer treatment through a uniquely supportive and positive lens, often with higher relationship satisfaction and reported self-image. As survivorship care continues to evolve, it is important to recognize not only the specific needs of sexual minority women, but also the many strengths of this population as these factors may inform future interventions and approaches to improved survivorship care.

## Figures and Tables

**Table 1:** Demographic characteristics of respondents (*n* = 15).

Variable	*n* = 15 (SD)	Straight Women (*n* = 10) (SD)	Lesbian Women (*n* = 5) (SD)
Mean age (years)	56.3 (10.86)	52 (6.870)	65 (12.116)
Mean age at diagnosis (years)	50.13 (8.66)	46.8 (4.686)	56.8 (10.703)
Demographic variables	*n* = 15 (%)	Straight Women (*n* = 10)(%)	Lesbian Women (*n* = 5)(%)
Sex at birth			
Male			
Female		10 (100)	5 (100)
Marital status			
Single	2 (13.3)	2 (20)	0
Married	6 (40)	5 (50)	1 (20)
Divorced	3 (20)	2 (20)	1 (20
Live in	4 (26.7)	1 (10)	3 (60
Household size			
1	3 (20)	2 (20)	1 (20)
2	11 (73.3)	7 (70)	4 (80)
3	0	0	0
4	1 (6.7)	1 (10)	0
Education			
No high school diploma	0	0	0
High school graduate	0	0	0
Some college	1 (6.7)	0	1 (20)
College graduate +	14 (93.3)	10 (100)	4 (80)
Race/ethnicity			
White, non-Hispanic	12 (80)	8 (80)	4 (80)
Black, non-Hispanic	1 (6.7)	1 (10)	
Employment status			
Employed full time	9 (60)	7 (70)	2 (40)
Unemployed, not seeking work	1 (6.7)	1 (10)	
Retired	3 (20)	1 (10)	2 (40)
Household annual income			
<$30,000	1 (6.7)	1 (10)	
$30,000–49,999	2 (13.3)	1 (10)	1 (20)
$50,000–74,999	3 (20)	2 (20)	1 (20)
>$75,000	7 (46.7)	5 (50)	2 (40)
Stage at diagnosis			
0	1 (6.67)		1 (20)
1	3 (20)	2 (20)	1 (20)
2	4 (26.7)	4 (40)	
3	5 (33.3)	4 (40)	1 (20)
4			
unknown	2 (13.3)		2 (40)
Type of breast cancer surgery			
Lumpectomy	5 (33.3)	3 (30)	2 (40)
Mastectomy	14 (93.3)	9 (90)	5 (100)
Type of breast reconstruction			
None	3 (20)	1 (10)	2 (40)
Immediate expander/implant	6 (40)	4 (40)	2 (40)
Immediate flap	1 (6.7)	1 (10)	
Delayed flap	2 (13.3)	2 (20)	
Delayed expander/implant	3 (20)	2 (20)	1 (20)
Chemotherapy?			
No	7 (46.7)	3 (30)	4 (80)
Yes, adjuvant	4 (26.7)	3 (30)	1 (20)
Yes, neoadjuvant	4 (26.7)	4 (40)	
Radiation?			
Yes	5 (33.3)	4 (40)	1 (20)
No	9 (60)	5 (50)	4 (80)
Health insurance status at diagnosis			
Insured from self	9 (60)	4 (40)	5 (100)
Insured from partner/spouse	6 (40)	6 (60)	

## Data Availability

The data presented in this study are available within the article.
